# The Institutional Worker

**Published:** 1912-06-22

**Authors:** 


### THE INSTITUTIONAL WORKER

**Being a Special Supplement to "The Hospital"**

### Editor's Notices.

**Contributions:**

Contributions should be written, or preferably typed, on one side of the paper only, and all articles sent in are accepted upon the distinct understanding that they are forwarded to THE HOSPITAL only.

The Editor cannot undertake to return MSS. not used, but when a stamped directed envelope is enclosed the MSS. may be returned if a special request is made.

Accepted articles and paragraphs of news will be paid for after publication at the scale rate.

**Address:**

To prevent delay all contributions and letters on editorial business must be addressed exclusively to the Editor, "The Hospital," 29 Southampton Street, Strand, London, W.C. It is important that this regulation shall be strictly observed.

**Correspondence:**

Correspondence on all subjects is invited. The name and address of correspondents must be given as a guarantee of good faith, but not necessarily for publication.

**Special Articles:**

Special articles are invited, and questions, inquiries, and paragraphs upon all matters relating to the work, administration, and management of general, special, mental, fever, cottage and convalescent hospitals, sanatoria, homes, institutions, societies and organisations for the treatment or care of the sick, injured and dependants of all classes. Every matter affecting the interests and welfare of the staff of all grades working in these institutions will receive special consideration.

**Administrative Medicine, Research and Items of News:**

Special terms will be paid for approved articles on subjects in this wide field by experts who have made some section of it their special study and interest. We wish to give prominence to every movement tending to advance accuracy of diagnosis, efficiency in treatment, and the development of modern methods to eradicate disease and promote the welfare of the suffering.

**A Bureau of Information:**

We invite inquiries and applications for information and help in providing for that numerous class of sufferers who require special care or house-room which they cannot obtain in their own homes or secure for themselves. **We cannot, however, prescribe, or recommend practitioners.**

**Books for Review:**

Publishers are particularly requested to send advance proofs of any new books of importance whenever possible, as the Editor has made arrangements to publish immediate reviews on a new plan.

**Photographs, Plans, Blocks, and Illustrations:**

It is requested that wherever possible MSS. may be accompanied by illustrations in any of the above forms. The name of the sender and of the article to which it belongs should be written on the back of each photograph, drawing, or block for purposes of identification.

**Local Papers:**

Newspapers containing reports or news paragraphs **should be marked** and addressed to the Sub-Editor.

**The Coupon System:**

A coupon will be found at the bottom of the third inside page of the cover each week. This coupon must be attached to every question or inquiry to which an answer is desired in THE HOSPITAL.

### Practical Points.

SINCE the extensive developments, which were inaugurated with the first issue of the New Volume of THE HOSPITAL in October last, the value of the Journal as a medium for the publication of all announcements which concern the interests of Institutional Workers and those in any way associated with Institutional Life has very materially increased. So rapidly have these notices grown in numbers that it was soon found necessary to introduce a Special Supplement, in order that they might secure a prominent position in the Journal. Consequently, "The Institutional Worker" became a permanent feature of THE HOSPITAL, and is unquestionably of the greatest service not only to the workers of the Institution, Health and Sanitary Services, but also to Institutional Authorities, since it enables them to secure adequate and effective publicity for such Vacancies as may occur from time to time on their Staffs. Moreover, as the only Journal dealing comprehensively with Institutional affairs, the circulation of THE HOSPITAL is of such a character as to render it of the greatest possible utility for all announcements in any way associated with Institutional Life and Work, and, indeed, it would be difficult to find a means that more effectually accomplishes the many purposes of its readers. Special columns are devoted to specific uses, as "Appointments Vacant," "Appointments Wanted," "Midwifery," "Medical and Nursing Homes," "Change of Air," "Sale and Exchange," &c., &c., and those of our readers who have not yet employed "The Institutional Worker" we would strongly recommend this section to their particular notice as a valuable and practical help in many directions.

### Manager's Notices.

**All letters on business matters must be addressed to The Manager, "The Hospital," 28 and 29 Southampton Street, London, W.C.**

**Advertisements:**

To ensure insertion, all advertisements for THE HOSPITAL must reach the Manager not later than Wednesday morning in each week. For scale of charges see pages v and 2.

**Subscriptions,** which may commence at any time, are payable in advance. Cheques and Post-Office Orders should be crossed London County and Westminster Bank, Covent Garden Branch, and made payable to the Manager of THE HOSPITAL.

RATES OF SUBSCRIPTION (including Postage).

| *United Kingdom.* | | *Foreign and Colonial.* | |
|---|---|---|---|
| Three Months | 2s. 0d. | Three Months | 2s. 6d. |
| Six Months | 4s. 0d. | Six Months | 5s. 0d. |
| Twelve Months | 6s. 6d. | Twelve Months | 8s. 8d. |

*SUBSCRIPTION FORM.*

*Please send me* THE HOSPITAL *for*..................*months, commencing with your issue of*.........................., *for which please find enclosed*............................

*Name*..................................................

*Address* ..................................................

..................................................

*Date*..................................

*To* .................................... *Newsagent.*

**Remittances:**

**It is especially requested that remittances be made by Cheque or Postal Order, and in halfpenny stamps only when the amount is under sixpence.**

### OFFICIAL APPOINTMENTS VACANT.

### Poor-Law Vacancies.

Assistant Cook (£22), Derby Union. Mr. N. Twigge, C. to G., Derby. June 27.
Assistant Female Cook (£26), Bermondsey Guardians. Matron of the Infirmary, Lower Road, Rotherhithe, S.E. June 26.
Assistant Male Attendant (£25), Plymouth Guardians. Mr. W. H. Davy, C. to G., Plymouth. June 24.
Assistant Matron (£45), Central London School District. Mr. G. P. Morrell, Clerk to the Managers, Hanwell, W. June 26.
Assistant Matron and Relief Assistant Matron (£20 each), Swansea Union. Mr. L. Jenkins, C. to G., Swansea. June 24.
Assistant Medical Officer (£220), Coventry Union. Mr. E. A. Evans, C. to G., Coventry. June 24.
Assistant Nurse (£25), Oxford Guardians. Master of the Workhouse, Oxford. June 25.
Assistant Nurse (£20), Dover Union. Mr. E. Carder, C. to G., Dover. June 26.
Assistant Nurse (£28), Eastry Union. Mr. F. S. Cloke, C. to G., Sandwich. July 1.
Assistant Nurse (£30), West London Schools. Mr. F. G. Beeching, Clerk to the Managers, Ashford, Middlesex. July 1.
Assistant Schoolmistress (£90), West London District Schools. Mr. F. G. Beeching, Clerk to the Managers, Ashford, Middlesex. July 4.
Charge Male Day Attendant (£40), Wandsworth Union. Mr. F. W. Piper, C. to G., St. John's Hill, Wandsworth, S.W. July 3.
Charge Nurse (£28), Tonbridge Union. N. R. Stone, C. to G., 23 Church Road, Tunbridge Wells. June 27.
Charge Nurse (£40), Hull Guardians. Mr. R. H. Winter, C. to G., Hull. June 25.
Charge Nurse (£30), Loughborough Union. Mr. A. W. Jarratt, C. to G., Loughborough. June 25.
Charge Nurse (£35), South Shields Union. Mr. J. W. Coulson, C. to G., South Shields. June 26.
Charge Nurse (£30), Swansea Union. Mr. L. Jenkins, C. to G., Swansea. July 2.
Cook (£25), Lincoln Union. Mr. W. B. Danby, C. to G., Lincoln. June 24.
Cook-General (£25), Gloucester Union. Mr. H. A. Armitage, C. to G., Gloucester. June 24.
Day Gate Porter (£30), Wandsworth Union. Mr. F. W. Piper, C. to G., St. John's Hill, Wandsworth, S.W. July 3.
Female Cook (£40), Bermondsey Guardians. Matron of the Infirmary, Lower Road, Rotherhithe, S.E. June 26.
Female Supernumerary Attendant (£25), Woolwich Union. Mr. T. Cutter, C. to G., Woolwich. June 26.
First Assistant Clerk (£150), Newcastle-on-Tyne Union. Mr. G. Walker, C. to G., Newcastle-on-Tyne. June 25.
Foster-Mother (£25), Christchurch Union. Mr. A. Druitt, C. to G., Christchurch. June 28.
Foster-Mother (£28), Brentford Union. Mr. W. Stephens, C. to G., Isleworth, W. July 2.
Foster-Mother (£25), Nuneaton Union. Mr. C. Blakeway, C. to G., Nuneaton. June 24.
Foster-Mother (£24), Coventry Union. Mr. E. A. Evans, C. to G., Coventry. June 24.
Foster-Mother (£20), Rochford Union. Mr. F. Gregson, C. to G., Southend-on-Sea. June 24.
Foster-Mothers (£26), West Ham Union. Mr. T. Smith, C. to G., Leytonstone, N.E. July 4.
Head Male Lunatic Attendant (£40), Birkenhead Union. Mr. J. Carter, C. to G., Birkenhead. June 25.

### Books for Institutional Workers.

"Midwife's Pocket Dictionary, with revised Rules of the C.M.B." ... ... ... ... 1s. 1d.
"The Nurses' Pronouncing Dictionary" ... ... 2s. 0d.
"A Handbook for Nurses." (J. K. Watson, M.D.) 5s. 4d.
"Art of Feeding the Invalid." ... ... ... ... 1s. 6d.
"Surgical Ward Work and Nursing." ... ... ... 5s. 5d.
"Massage Movements, including the Nauheim Exercises" ... ... ... ... ... ... 1s. 1d.
"Surgical Instruments and Appliances used in Operations" (Harold Burrows, M.B.London, B.S., F.R.C.S.) ... ... ... ... ... ... 1s. 8d.
"The Nurse's Materia Medica" (Herbert French, M.D.) ... ... ... ... ... ... ... 2s. 9d.
Case-papers, Charts, Professional Cards, and every kind of Institutional Literature.

Of all Booksellers or of The Scientific Press, Limited, 28 and 29 Southampton Street, Strand, London, W.C.

